# Local discrepancies in continental scale biomass maps: a case study over forested and non-forested landscapes in Maryland, USA

**DOI:** 10.1186/s13021-015-0030-9

**Published:** 2015-08-16

**Authors:** Wenli Huang, Anu Swatantran, Kristofer Johnson, Laura Duncanson, Hao Tang, Jarlath O’Neil Dunne, George Hurtt, Ralph Dubayah

**Affiliations:** 1 Department of Geographical Sciences, University of Maryland, College Park, USA; 2 USDA Forest Service, Northern Research Station, Newtown Square, PA USA; 3 Rubenstein School of the Environment and Natural Resources, University of Vermont, Burlington, USA

**Keywords:** Temperate deciduous forest, Lidar, Aboveground biomass, Carbon

## Abstract

**Background:**

Continental-scale aboveground biomass maps are increasingly available, but their estimates vary widely, particularly at high resolution. A comprehensive understanding of map discrepancies is required to improve their effectiveness in carbon accounting and local decision-making. To this end, we compare four continental-scale maps with a recent high-resolution lidar-derived biomass map over Maryland, USA. We conduct detailed comparisons at pixel-, county-, and state-level.

**Results:**

Spatial patterns of biomass are broadly consistent in all maps, but there are large differences at fine scales (RMSD 48.5–92.7 Mg ha^−1^). Discrepancies reduce with aggregation and the agreement among products improves at the county level. However, continental scale maps exhibit residual negative biases in mean (33.0–54.6 Mg ha^−1^) and total biomass (3.5–5.8 Tg) when compared to the high-resolution lidar biomass map. Three of the four continental scale maps reach near-perfect agreement at ~4 km and onward but do not converge with the high-resolution biomass map even at county scale. At the State level, these maps underestimate biomass by 30–80 Tg in forested and 40–50 Tg in non-forested areas.

**Conclusions:**

Local discrepancies in continental scale biomass maps are caused by factors including data inputs, modeling approaches, forest/non-forest definitions and time lags. There is a net underestimation over high biomass forests and non-forested areas that could impact carbon accounting at all levels. Local, high-resolution lidar-derived biomass maps provide a valuable bottom-up reference to improve the analysis and interpretation of large-scale maps produced in carbon monitoring systems.

**Electronic supplementary material:**

The online version of this article (doi:10.1186/s13021-015-0030-9) contains supplementary material, which is available to authorized users.

## Background

Accurate maps of forest aboveground biomass are critical for reducing uncertainties in the carbon cycle and informing carbon management decisions [1–3]. While no method provides direct measurements of biomass over large scales, a combination of remotely sensed data and a well established field inventory is considered suitable for monitoring programs such as REDD+ [4, 5]. Data inputs for biomass estimation have varied widely with tradeoffs between availability, cost and coverage. Accuracy of estimated biomass has also varied with the sensitivity of data to forest structure, spatial resolution, choice of statistical models, and the accuracy of field training data. Regardless, biomass estimates from different maps seem to agree at very coarse scales [4]. For example, Mitchard et al. [4] found that pan-tropical biomass maps converged at regional scales even though they varied locally. They concluded that uncertainties were largely related to spatial patterns of forest cover change. Langner et al. [5] evaluated pan-tropical biomass maps and successfully combined them into a framework for deriving REDD+ Tier 1 carbon storage estimates. While these findings are encouraging for national and continental scale reporting, there is a need to examine local discrepancies more closely as errors or uncertainty at fine-scales can complicate the use of coarse scale maps in local planning and decision making.

Almost all large-area biomass maps are derived from two-dimensional remote sensing data that have wide coverage but are generally less sensitive to canopy structure, particularly in moderate to high biomass forests (e.g. multispectral and single polarized SAR). Furthermore, they do not currently include fine scale variations in tree cover because of their coarse spatial resolution. Lidar instruments measure three-dimensional canopy structure which improves the accuracy of biomass maps [3] but lidar datasets have limited coverage and are expensive to acquire. An alternative is to use high-resolution lidar derived biomass maps, where available, to evaluate existing coarse scale maps, and make them more compatible for decision-making.

In 2010, NASA initiated the Carbon Monitoring System (CMS) to quantify carbon sources and sinks for an improved understanding of the global carbon cycle [6]. The program combines top-down continental scale approaches with bottom-up local scale approaches. The top-down approach relies on satellite observations to quantify carbon storage and terrestrial fluxes for national reporting. The bottom-up approach focusses on mapping carbon stocks and uncertainties at fine scales. Within the US, continental scale maps use Forest Inventory Analysis (FIA) plot data for model development, and biomass estimates are in turn compared with FIA county or regional averages as a type of validation [7–9]. However, these validations are not based on independent data, and often lack constraints at high spatial resolution. Moreover, field inventories generally do not include trees outside forests [9]. Continental scale maps therefore do not predict biomass outside forested areas and may significantly underestimate carbon balances [10].

A thorough understanding of local-scale discrepancies requires an independently derived high-resolution estimate. Recently, such a map was produced for the state of Maryland as part of CMS [11, 12]. Biomass estimates were derived from lidar data in conjunction with non-FIA field data using machine-learning approaches. The 30 m biomass maps incorporated tree canopy cover at the 1 m resolution, thus including forested and non-forested trees in the process. This local scale effort provides a reference for evaluating existing coarse scale maps.

We present results from a detailed comparison of the biomass map produced over Maryland (hereafter referred to as CMS_RF) with four national scale biomass maps: (A) NBCD2000 [13], (B) Blackard [14], (C) Wilson [15], and (D) Saatchi [16] at the pixel-, county- and state-level. We quantify the degree and spatial patterns of differences to gain an improved understanding of map discrepancies and their impacts on carbon accounting.

## Methods

### Study area and field data

**Fig. 1:**
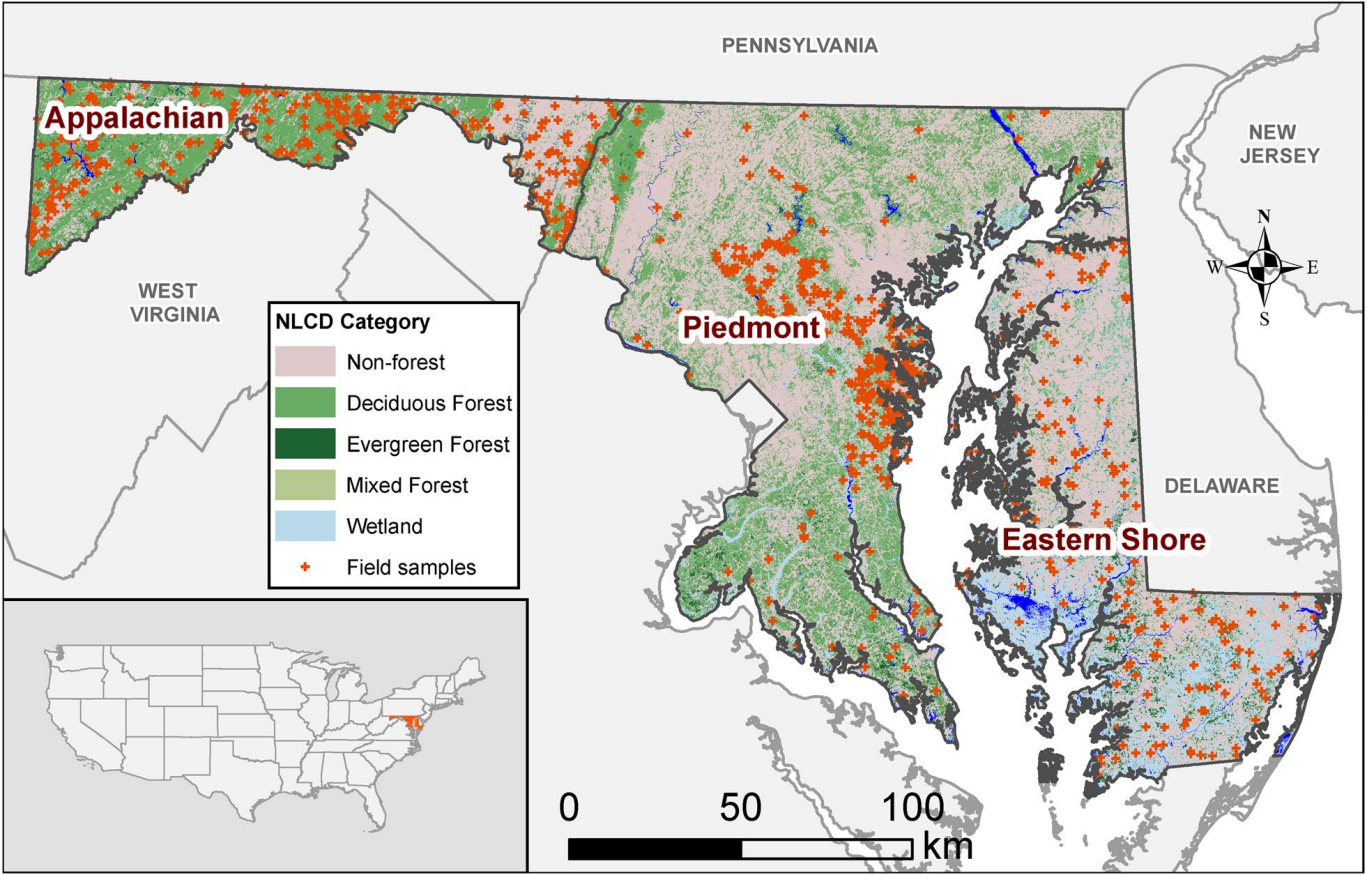
Study area showing physiographic regions and field plot locations. Physiographic provinces (*Appalachian*, *Piedmont*, and *Eastern Shore*) are divided based on species-composition and environmental gradients. Land cover classes (*Evergreen*, *Deciduous*, *Mixed*, *Wetlands*, and *Non*-*forest*) are taken from the NLCD2006 database.

Maryland has a land area of ~25,600 km^2^ (Fig. 1) and can be divided into 3 major physiographic provinces (or ecoregions) based on species-composition and environmental gradients. These are the Eastern Coastal Plain (hereafter, “Eastern Shore”), the combined Western Coastal Plain and Piedmont (hereafter, “Piedmont”) and the combined Blue Ridge, Valley and Central Appalachians (hereafter, “Appalachian”). The wide variability in topography, forest types, and environmental gradients makes it a suitable test-bed for national map comparisons.

We first generated a biomass map using existing lidar data and independent field estimates. Field data were collected in 848 variable and fixed radius plots selected through a stratified sampling of NLCD land cover (evergreen, deciduous, wetlands, mixed and non-forest) and lidar canopy heights (Fig. 1). Tree measurements of diameter at breast height (dbh) and species were recorded in each plot. Allometric estimates of aboveground biomass (Mg ha^−1^) were calculated for each tree using equations from Jenkins et al. [17] and appropriate blow up factors were applied to estimate biomass density for the variable radius plots. For more details on field data collection, refer to [11, 18]. In addition to these new plots, FIA data were obtained from across the state and used for model validation only [9].

### Local scale CMS_RF biomass map

Leaf-off, discrete return lidar data were obtained from the Maryland Department of Natural Resources (DNR) and individual counties. Tree canopy cover and canopy height were mapped at 1 m resolution using a combination of Lidar and high-resolution leaf-on multispectral imagery for every county and seamlessly across the entire state [19, 20]. Lidar canopy height models were masked using high-resolution tree cover to obtain canopy heights over forested and non-forested areas. Lidar metrics such as height percentiles, densities, and canopy cover were calculated within 30 m grid cells corresponding to the NLCD land cover dataset. Field based estimates of biomass were then related to the lidar metrics using Random Forests regression models [21, 22]. Three separate empirical models were developed, one for each physiographic region, and were applied to predict biomass for counties within the region. Predictions over individual counties were merged into a statewide biomass map at 30 m resolution (CMS_RF map). Details of the biomass estimation are available in [18] and [12].

### Continental scale biomass maps

**Fig. 2:**
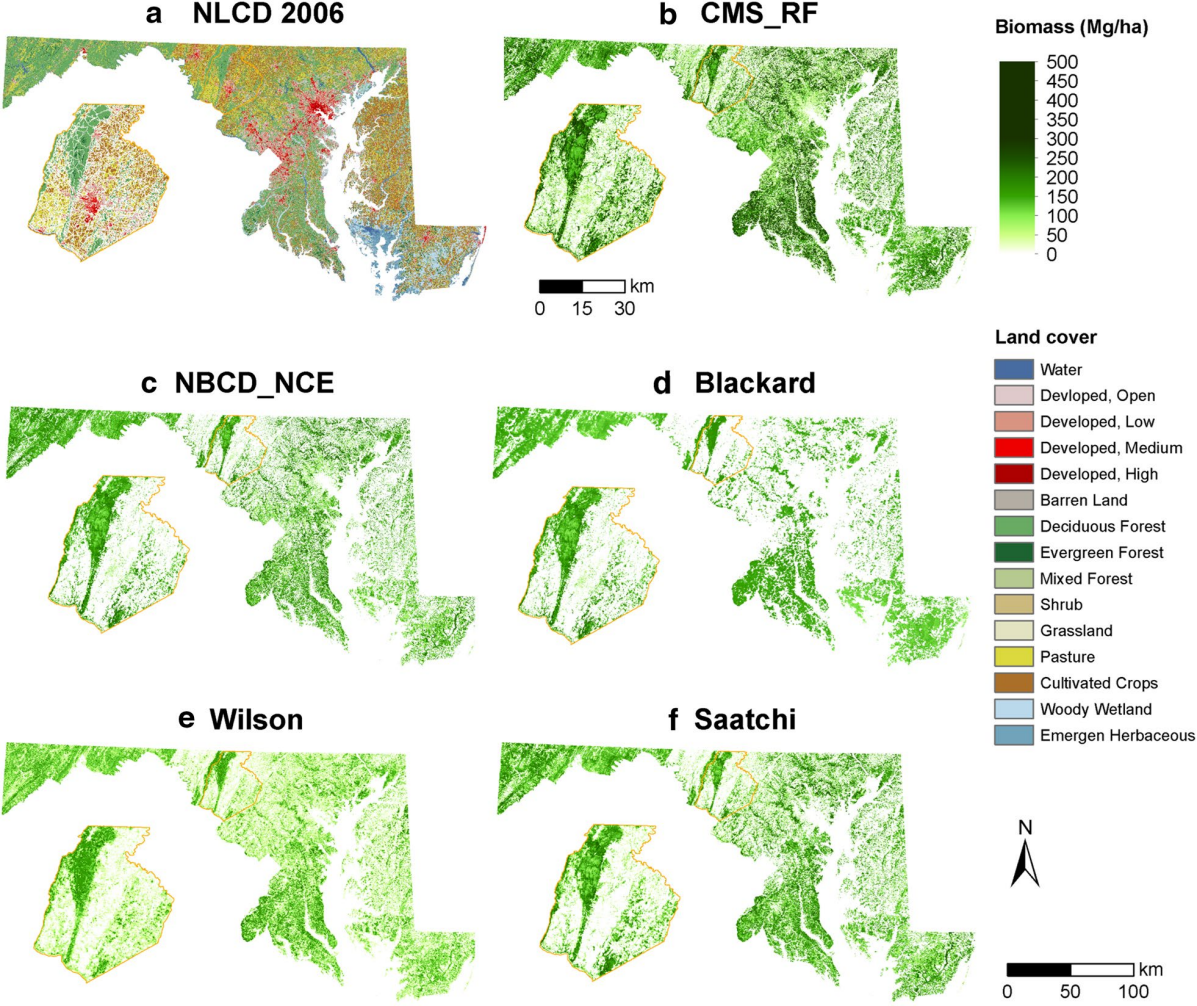
Biomass density maps at the state level. **a** Land cover at 30 m spatial resolution; **b** CMS_RF biomass product at 30 m spatial resolution; **c** NBCD_NCE biomass product at 30 m spatial resolution; **d** Blackard biomass product at 250 m spatial resolution; **e** Wilson biomass product at 250 m spatial resolution; and **f** Saatchi biomass product at 100 m spatial resolution.

Four national biomass products (Fig. 2; Table 1) were compared to the CMS_RF map. Each of these maps was derived using medium to coarse resolution satellite imagery. The NBCD2000 was the first 30 m national product developed using InSAR data from the 2000 Shutter Radar Topography Mission (STRM) and Landsat ETM+ data [13, 23]. NBCD2000 provided two versions of biomass: (A) NBCD_FIA map in which tree-level biomass estimates were obtained from tree tables in the FIA database (FIADB); and (B) NBCD_NCE or National Consistent allometric Equations in which biomass estimates were derived from equations developed by Jenkins et al. [17]. We used the NBCD_NCE version for consistency with our field biomass estimates, which were also derived from national allometric equations.

**Table 1:** Summary of biomass products used in this study

Product	Sensor and year	Field data and year	Resolution	Forest mask	Approach	Uncertainty map	References
CMS_RF	DRL 2004–2012	2011–2014	30 m	NAIP high-res tree canopy cover	Random forest, regression tree	Percentile error (QRF)	[12, 18]
NBCD_NCE	Landsat + SRTM 2000	2000	30 m	NLCD 2001	Random forest, regression tree	Quality voids	[13]
Blackard	MODIS 2001^a^	2005–2009	250 m	NLCD 1992	Cubist, regression tree	Relative error	[14]
Wilson	MODIS 2002–2008	2005–2009	250 m	NLCD 2001 percent tree canopy 25 %	PGNN, kNN		[15]
Saatchi	MODIS + PALSAR + Landsat	2005	~250 m v1^b^ ~90 m v2^b^	NLCD 2006	MaxEntropy, parametric	Percent error	[16]

^a^Year is national maps in eastern US.

^b^Original maps are in lat/lon, where v1 with 0.00222222 ≅ 250 m and v2 with 0.00083333 deg ≅ 90 m.

*DRL* Discrete Return Lidar, 1–2 m small footprint lidar aggregate, *NAIP* National Agriculture Imagery Program, *QRF* Quantile Regression Forests, *SRTM* Shuttle Radar Topography Mission, *PALSAR* Phased Array type L-band Synthetic Aperture Radar, *PGNN* Phenological Gradient Nearest Neighbor, *kNN* k-nearest neighbor.

The Blackard map was developed at the 250 m spatial resolution [14] using tree-based regression (i.e., Cubist). It was developed by relating FIA plot data to multi-variable geospatial predictors, including Moderate Resolution Imaging Spectrometer (MODIS) data in 2001, percent tree cover and land cover proportions (from the NLCD 1992 product), topographical variables, and annual climate parameters, etc.

The Wilson map, also developed at the 250 m spatial resolution, was derived from MODIS imagery data from 2002 to 2008 and FIA field plots using a Phenological Gradient Nearest Neighbor (PGNN) imputation approach and canonical correspondence analysis (CCA) models [15, 24]. The Wilson map is a newer and improved version of the Blackard map.

The Saatchi map is a CMS national-scale map derived using a combination of NASA remote sensing data, forest inventory and ancillary data (the same method as [25]). Waveforms from the Geoscience Laser Altimeter System (GLAS) lidar were used to derive Lorey’s height, which was then related to FIA biomass. The GLAS shots with predicted biomass estimates were used as ground truth (i.e., biomass plot samples) and related to multiple remote sensing inputs, including MODIS, PALSAR, and Landsat imagery using Maximum Entropy (MaxEnt) models for predicting biomass at the continental scale. An updated version of the Saatchi map (Saatchi et al., personal communication) reported improvements such as: (A) reprocessed GLAS data, (B) 15 allometric equations that include three forest types (deciduous, coniferous, and mixed) for 5 regions of the US, and (C) NLCD non-vegetated gaps filled by PALSAR and Landsat data. We present results from the updated version but also include a comparison of the old and new versions in the supplement (Additional file 1: Figure S1 and Additional file 2: Figure S2).

### Map comparisons

All maps were warped to a common frame of reference (UTM 18N NAD 83) ensuring minimum distortion to the native projections. Maps were matched to the same extents and pixel sizes. The 30 m biomass density maps (e.g. CMS_RF and NBCD_NCE) were aggregated to 250 m and coarser resolutions (e.g. 500 m, 1 km, and 4 km). The Wilson, Blackard (originally 250 m), and Saatchi maps (originally ~90 m) were each aggregated to 500 m, 1 km, and 4 km.

A canopy cover mask was used to differentiate between forested and non-forested areas in our comparisons. The mask was created from the NLCD 2006 dataset for consistency with the land cover used in the CMS_RF stratification [18] and [12]. The mask included deciduous forest (41), evergreen forest (42), mixed forest (43), woody wetlands (90) and emergent herbaceous wetlands (95) from the NLCD dataset. NLCD defines forest as more than 20 percent of areas dominated by trees. Therefore, a 20 % threshold was set while aggregating the mask from 30 to 250 m and other coarse resolutions. Comparisons were made over: forested areas only; non-forested areas only; and over forested and non-forested areas combined.

Statistical indicators such as coefficient of determination (R^2^), root mean squared difference (RMSD), RMSD% or CV (coefficient of variation of the RMSD), and mean bias error (MBE) were used to compare the CMS_RF product with the four national maps. The Fuzzy Numerical Index (FNI) is a valuable quantitative descriptor of the spatial similarities and differences between maps and was included in our comparisons, following [26].

1 $$R^{2} = 1 - \frac{{\sum\nolimits_{i = 1}^{n} {(C_{i} - M_{i} )^{2} } }}{{\sum\nolimits_{i = 1}^{n} {(M_{i} - \overline{M} )^{2} } }}$$

2 $$RMSD = \sqrt {\sum\limits_{i = 1}^{n} {\frac{{(C_{i} - M_{i} )^{2} }}{n}} }$$

3 $$RMSD\% = \frac{RMSD}{{\overline{C} }} \times 100$$

4 $$MBE = \frac{{\sum\nolimits_{i = 1}^{n} {(C_{i} - M_{i} )} }}{n}$$

5 $$FNI = \frac{{\sum\nolimits_{i = 1}^{n} {1 - \frac{{\left| {C_{i} - M_{i} } \right|}}{{\hbox{max} (C_{i} ,M_{i} )}}} }}{n}$$

*M*_*i*_ is the value of national map; *C*_*i*_ is the CMS_RF predicted value; *i* is the sample index; $\overline{C}$ and $\overline{M}$ are the means of CMS_RF and national map respectively; and *n* is the sample size.

### Results

Spatial patterns of biomass were consistent with land cover and physiographic gradients in visual comparisons. Within forested areas, all maps showed distinct dendritic patterns corresponding to riparian zones that had higher biomass than surrounding areas. Similar spatial patterns of biomass were also noted along ridges, valleys and forested patches with high structural variability.

**Fig. 3:**
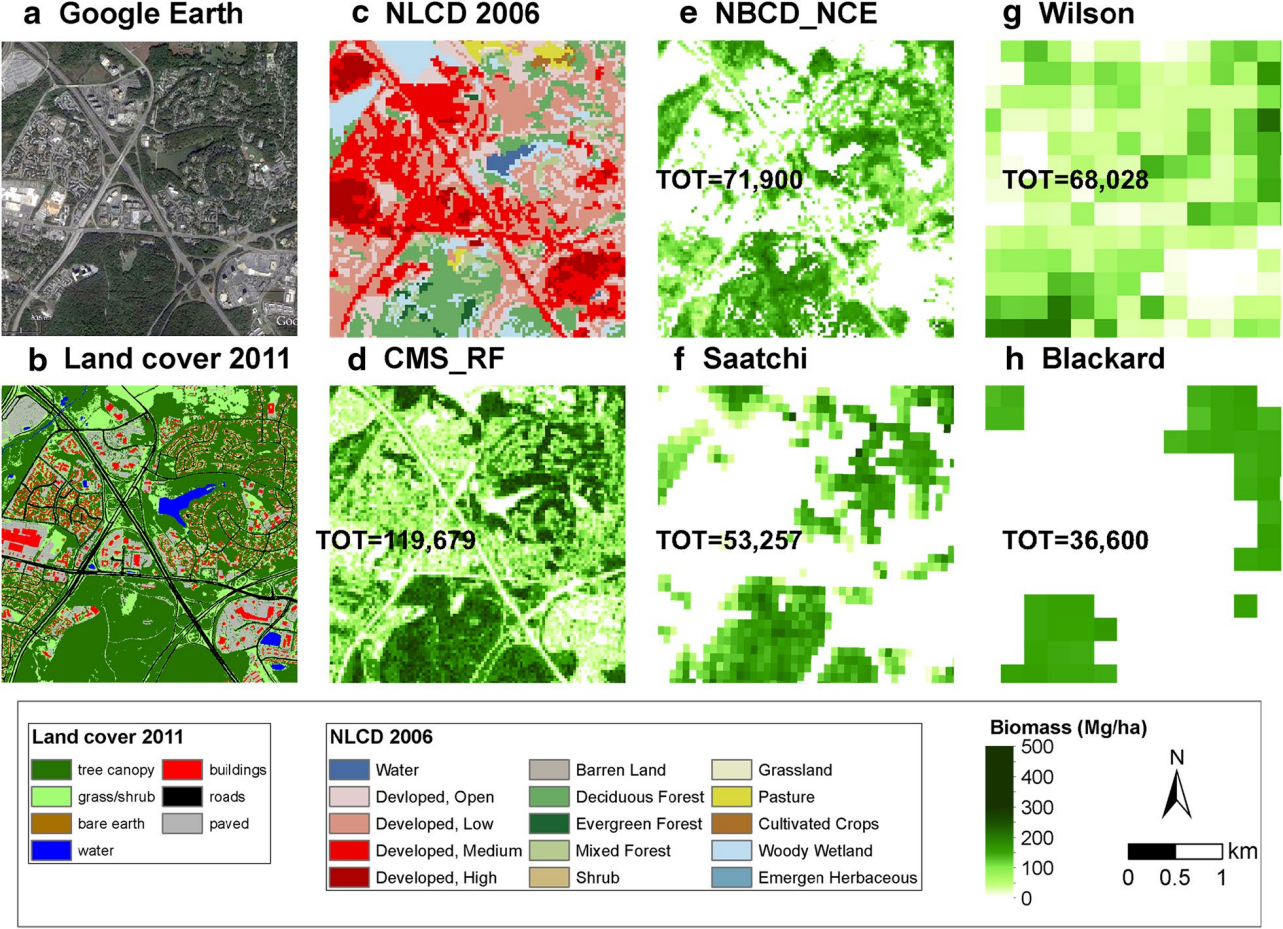
Discrepancies in spatial distribution of biomass density at fine-scale. **a** Google Earth image in 2012; **b** high resolution [1 m] land cover map; **c** NLCD2006; **d** CMS_RF biomass product at 30 m spatial resolution; **e** NBCD_NCE biomass product at 30 m spatial resolution; **f** Saatchi biomass product at 100 m spatial resolution; **g** Wilson biomass product at 250 m spatial resolution; and **h** Blackard biomass product at 250 m spatial resolution. Zoom-in figures are for Frederick County.

Although spatial patterns were similar, biomass densities and levels of detail varied considerably (Fig. 2). The CMS_RF biomass map provided greater detail over urban/suburban landscapes (Fig. 3, e.g. trees along roadsides, hedges and backyards) when compared visually with high-resolution [1 m] land cover map and high-resolution imagery (Google Earth). The other maps predicted little or no biomass in non-forested areas. Differences over heterogeneous areas were particularly large (Fig. 3). Results ranged between 36,600 and 119,679 Mg, showing wide local-scale differences.

**Fig. 4:**
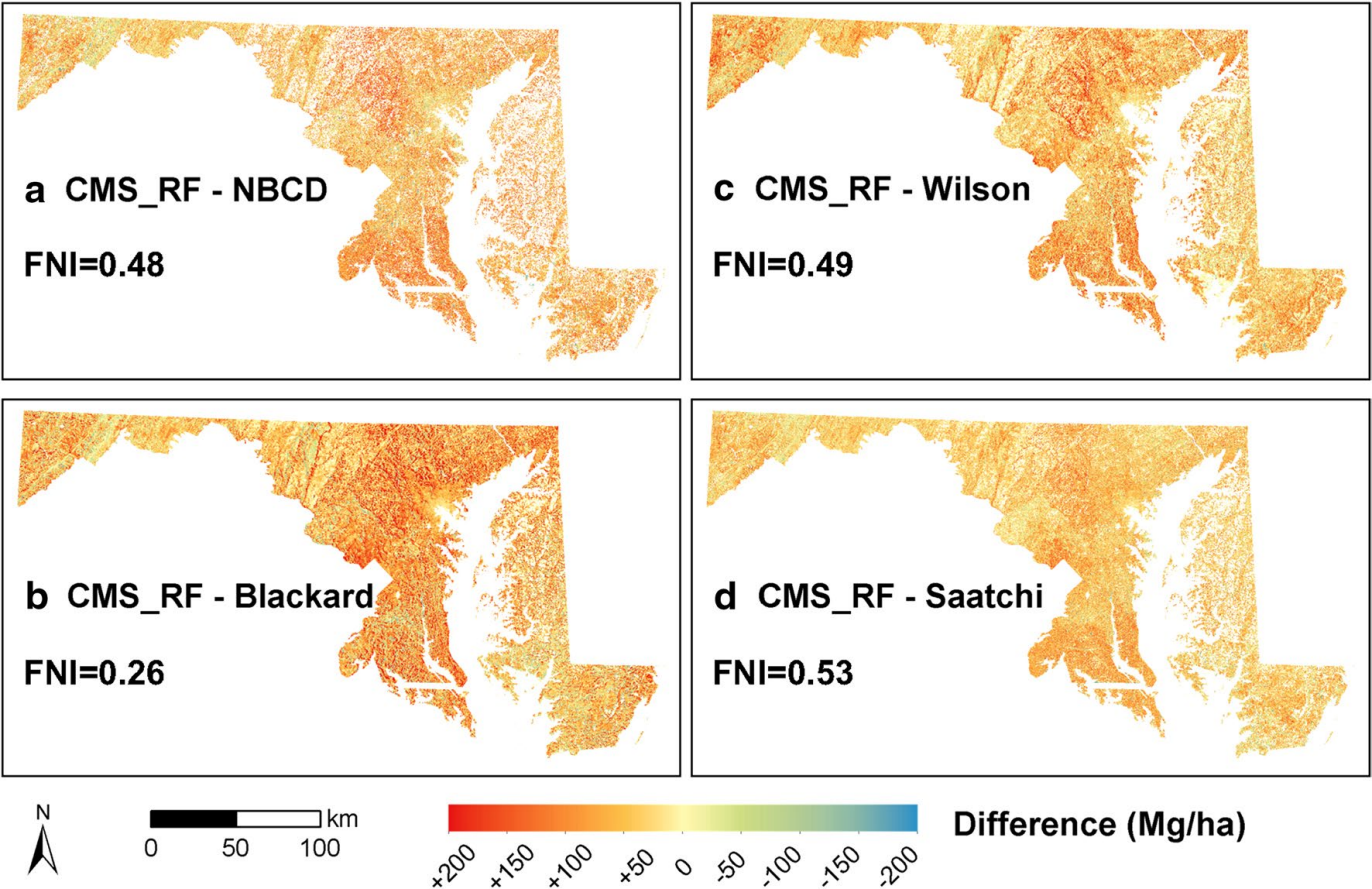
Difference maps of biomass density. **a** CMS_RF-NBCD_NCE at 30 m spatial resolution; **b** CMS_RF-Blackard at 250 m spatial resolution; **c** CMS_RF-Wilson at 250 m spatial resolution; and **d** CMS_RF-Saatchi at 100 m spatial resolution. Areas in *red* have lower values and areas in *blue* have higher values than the CMS_RF map. Fuzzy Numerical Index (*FNI*) quantifies overall similarity between the national biomass maps and the CMS_RF map, ranging from 0 (fully distinct) to 1 (fully identical).

FNI provides a spatial representation of similarities and differences when calculated at a pixel-level. However, it does not capture the positive and negative deviations with respect to the CMS_RF map. We therefore calculated a mean FNI value for each map comparison with values ranging from 0 (perfect dissimilarity) to 1 (perfect similarity). A combination of map differences and FNI index values provided additional spatial and quantitative understanding of map discrepancies (Fig. 4; Table 2). Differences between maps were prominent in the Piedmont region, over counties in southern Maryland and along the Appalachians in the West. The Saatchi map was most similar to the CMS_RF map (FNI = 0.53) while the Blackard Map (FNI = 0.26) was the most dissimilar. The Wilson map had almost an equal proportion of similar and dissimilar pixels (FNI = 0.49) while the NBCD map was slightly lower with an FNI of 0.48.

**Table 2:** Mean Fuzzy Numeric Index

Name	All	Forest	Non-forest
NBCD_NCE	0.48	0.62	0.22
Blackard	0.26	0.38	0.04
Wilson	0.49	0.52	0.41
Saatchi	0.53	0.69	0.25

Values calculated from maps at 250 m resolution.

### Comparisons at the pixel level

**Fig. 5:**
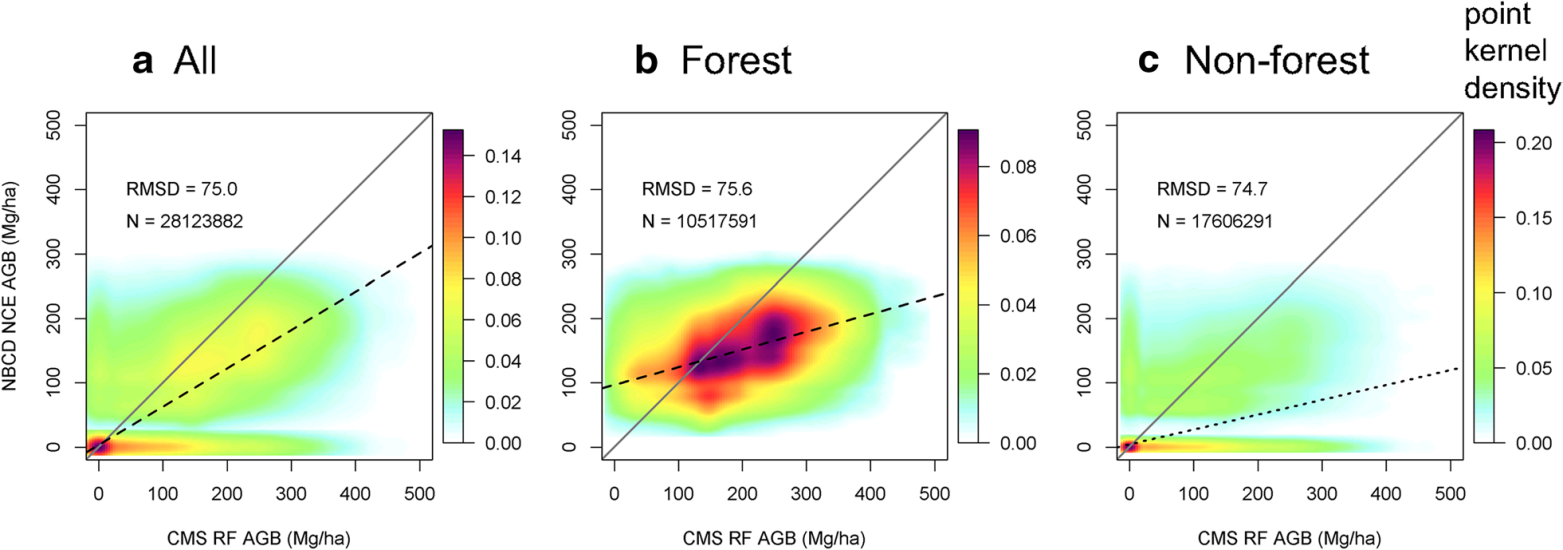
Scatter plots of NBCD_NCE biomass product versus CMS_RF biomass product (30 m) at state-level. **a** All; **b** forest; and **c** non-forest. The *x* axis in each plot represents biomass values from CMS_RF, and the *y* axis represents the biomass values from NBCD_NCE product. *N* is the number of pixel used in calculation of RMSD. *Black dashed line* is the fitted regression lines, *gray solid line* is the 1:1 line, and *light blue* to *dark red colors* represent point kernal density. Forest and non-forest categories are derived from NLCD2006 dataset at 30 m spatial resolution.

(i) High-resolution comparisons [30 m]
  Pixel level comparisons between the NBCD_NCE and CMS_RF biomass products showed wide scatter with a large number of zero biomass predictions from the NBCD map (Fig. 5). Most areas that did not have biomass values on the NBCD map had predictions in the CMS_RF map. The NBCD biomass values were biased lower than the 1:1 line with an overall RMSD of 75.0 Mg ha^−1^.

**Fig. 6:**
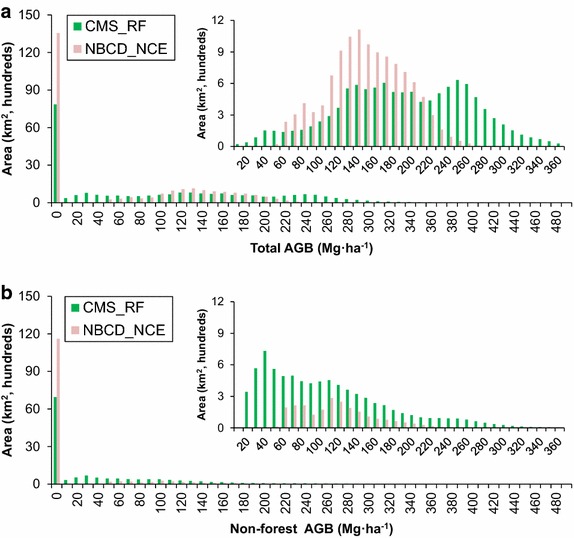
*Histograms* showing the biomass distribution of CMS_RF and NBCD_NCE products over the state of Maryland at 30 m resolution in 10 Mg ha^−1^ bins. **a** All and **b** non-forest. Note that zero values are ignored in the *inset* plots. Non-forest category is derived from NLCD2006 dataset.

**Fig. 7:**
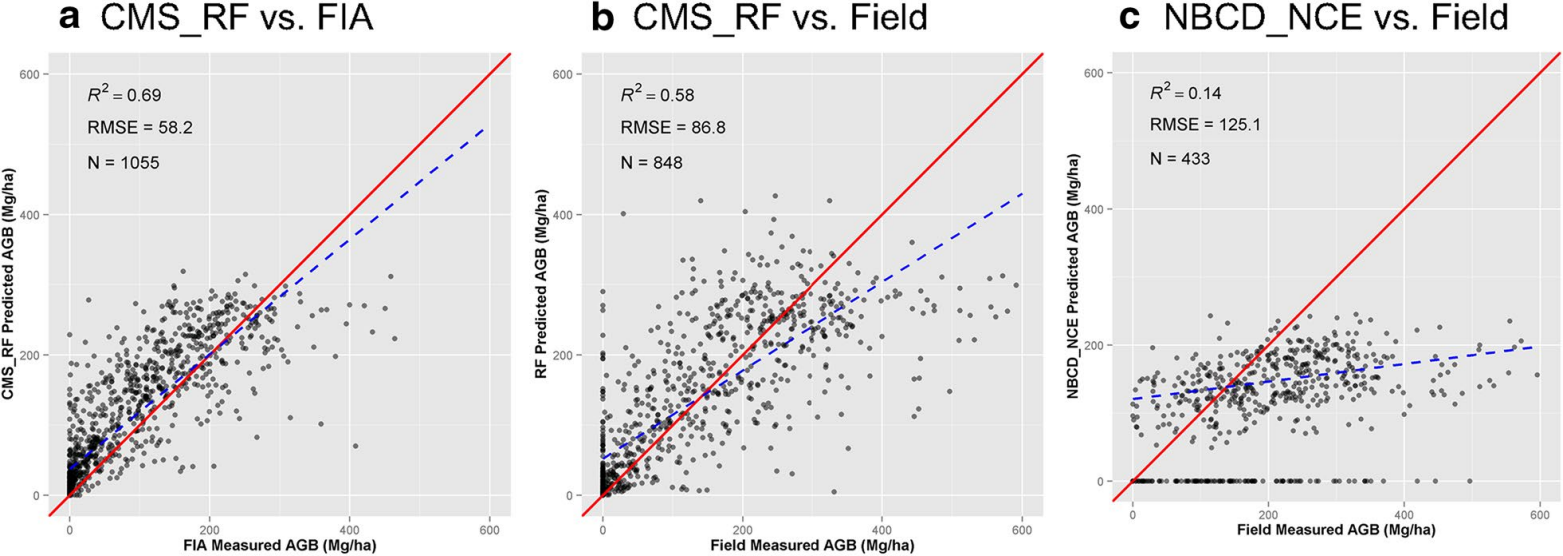
Scatter plots of CMS_RF and NBCD_NCE biomass products against FIA plots and CMS field plots. **a** CMS_RF vs. FIA, **b** CMS_RF vs. Field, and **c** NBCD_NCE vs. Field. The *red solid line* is the 1:1 line. The *blue dashed line* is the fitted regression with the filtered dataset, which exclude zero biomass in NBCD_NCE data. R^2^ and RMSD are calculated based on the filtered dataset.

**Fig. 8:**
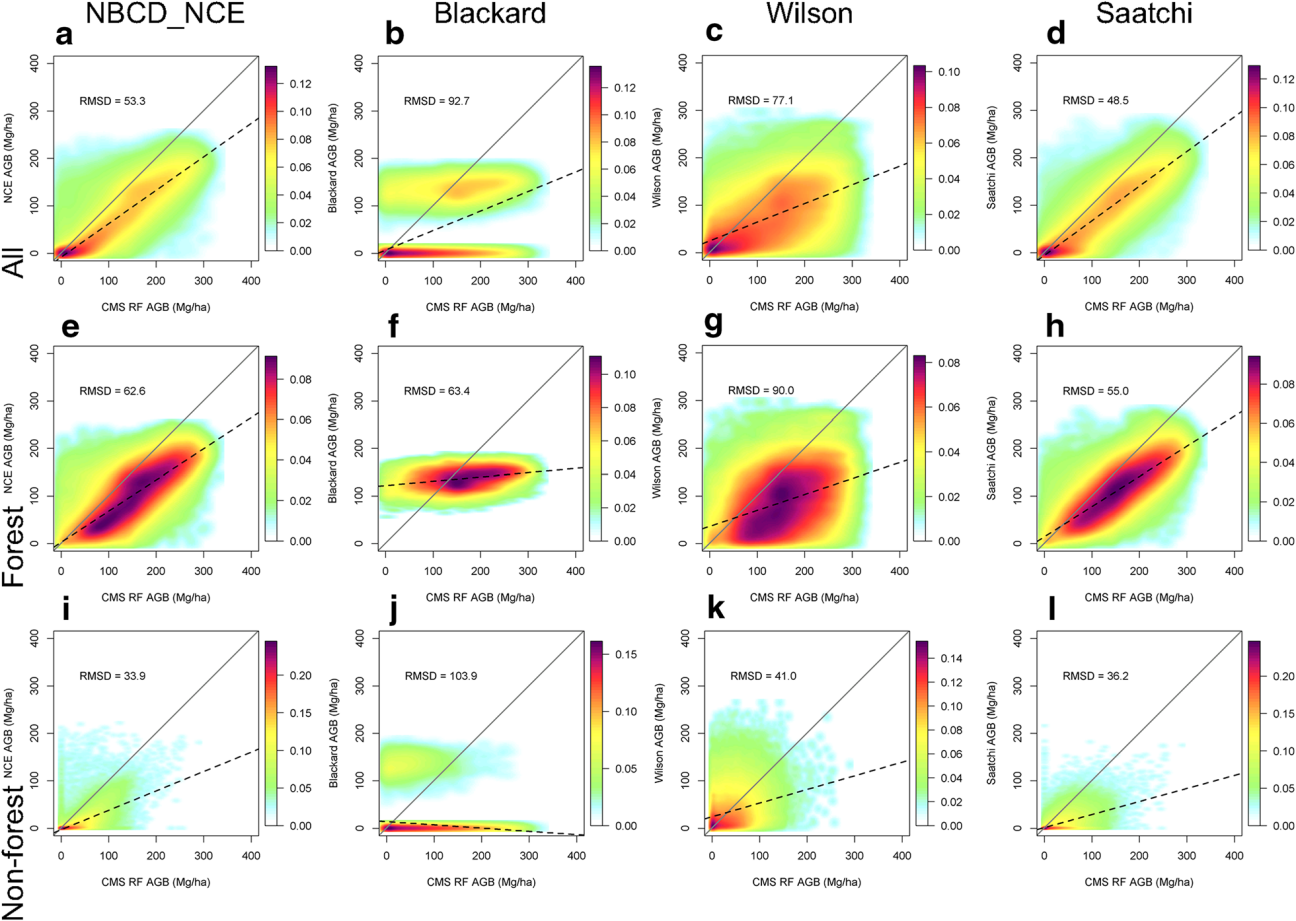
Scatter plots of biomass density at 250 m resolution from four national products versus CMS_RF product. From *left* to *right* are NBCD_NCE, Blackard, Wilson, and Saatchi, respectively. From *top* to *down* are NLCD2006 categorized total, forest, and non-forest, respectively. The *y* axis in each plot represents biomass values from national products, and the *x* axis represents the biomass values from CMS_RF product. Black dashed line is the fitted regression lines, *gray solid line* is the 1:1 line, and *light blue* to *dark red* represents sample kernal density. Forest and non-forest category are derived from aggregated NLCD2006 dataset at 250 m spatial resolution, with a threshold of 20 percentage for forest.

Biomass distributions from CMS_RF and NBCD_NCE maps showed large differences over total and non-forested regions (Fig. 6). The NBCD_NCE distribution was bimodal with modes shifted toward the left (or lower biomass ranges). The CMS_RF map had higher and more widely distributed values over non-forested regions. The NBCD_NCE dataset did not predict biomass outside forests but the non-forest histograms had some high biomass values. This was because an older NLCD (2000) forest/non-forest mask was used to generate the NBCD_NCE map. The time lag between the maps and the difference in forest/non-forest masks complicated the comparisons but did not affect the overall trend in the forested and non-forested scatter plots (Fig. 5).

(ii) Comparisons with field data
  We compared predictions from the CMS_RF and NBCD_NCE maps with biomass estimates from FIA data (average of four sub-plots) (Fig. 7a) and our variable radius field plots (Fig. 7b, c). The Random Forests model used to generate the CMS_RF map explained ~50 % variability in biomass from variable radius field plots (R^2^ = 0.49, RMSE = 89.3 Mg ha^−1^, n = 848). A cross-validation of the CMS_RF map with plot level FIA data showed higher agreement, partly due to higher sample number (R^2^ = 0.69, RMSE = 58.2 Mg ha^−1^, n = 1,055). On the other hand, a cross validation of the NBCD_NCE map with variable radius estimates resulted in substantially weaker relationships (R^2^ = 0.14, RMSE = 125.1 Mg ha^−1^, n = 433).

(iii) Comparisons at the pixel level [250 m]
  Large disagreements were observed in the scatter plots and associated errors at the 250 m resolution (Fig. 8). Overall RMSD values ranged between 48.5 and 92.7 Mg ha^−1^. The RMSD values ranged between 55.0 and 90.0 Mg ha^−1^ over forested regions, and between 33.9 and 103.9 Mg ha^−1^ over non-forested regions. The Saatchi and NBCD maps agreed more closely with the CMS_RF map with fewer zero biomass values after spatial aggregation. The updated version of Saatchi map agreed closely with the NBCD and CMS_RF map, while the original version showed a large difference (Additional file 1: Figure S1 & Additional file 2: Figure S2). The Blackard map was the least correlated with the CMS_RF map while the Wilson map had a large scatter around the 1:1 line.

**Fig. 9:**
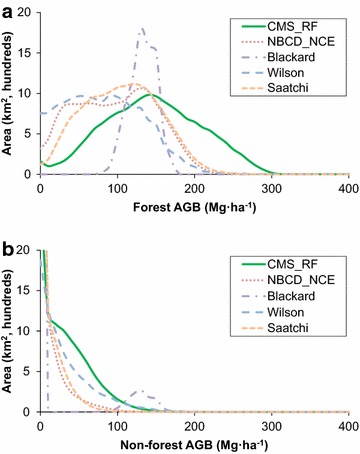
*Histograms* showing the biomass density distributions of CMS_RF, NBCD_NCE, Blackard, Wilson, and Saatchi products over the state of Maryland at 250 m resolution in 10 Mg ha^−1^ bins. **a** Forest, and **b** non-forest.

Histograms of biomass in intervals of 10 Mg ha^−1^ were generated and analyzed over the entire range (0–400 Mg ha^−1^) (Fig. 9). There was little agreement among the maps across the entire range of predicted values. The only similarities were between the NBCD_NCE and the Saatchi map above 125 Mg ha^−1^. The distribution of biomass in different ranges was also vastly different. Biomass values in the Blackard map were predominantly between 100 and 150 Mg ha^−1^ while all other datasets had values less than 250 Mg ha^−1^. Only the CMS_RF maps had predictions in ranges greater than 250 Mg ha^−1^.

### Comparisons at the county level

**Fig. 10:**
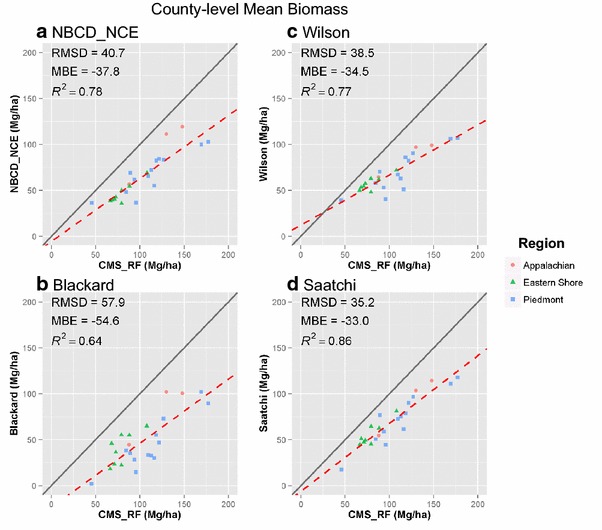
Scatter plots of county-level mean biomass density of four national products versus CMS_RF product. **a** NBCD_NCE at 30 m spatial resolution, **b** Blackard at 250 m spatial resolution, **c** Wilson at 250 m spatial resolution, and **d** Saatchi at 250 m spatial resolution. The *x* axis represents the biomass density values from CMS_RF product and the *y* axis represents corresponding national products in each plot. *Gray solid line* is the 1:1 line, and *red dashed line* is the fitted regression line.

**Fig. 11:**
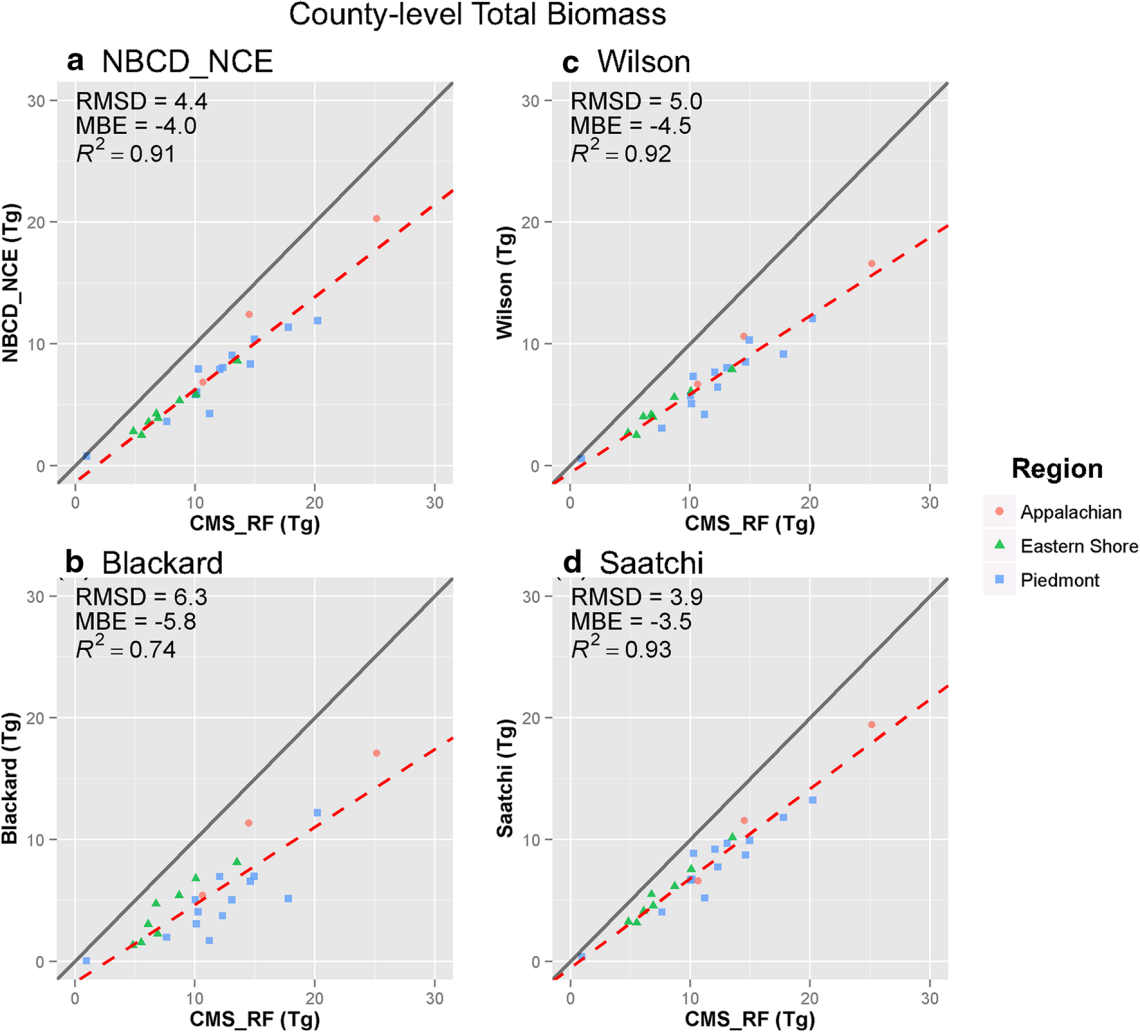
Scatter plots of county-level total biomass of four national products versus CMS_RF product. **a** NBCD_NCE at 30 m spatial resolution, **b** Blackard at 250 m spatial resolution, **c** Wilson at 250 m spatial resolution, and **d** Saatchi at 250 m spatial resolution. The *x* axis represents the values from represents biomass values from CMS_RF product and the *y* axis represents corresponding national products in each plot. *Red dashed line* is the fitted regression line, and *gray solid line* is the 1:1 line.

At the county level, the four maps showed improved correlation with the CMS_RF map in both mean (Fig. 10) and total biomass (Fig. 11). Among the three physiographic regions, the counties in Appalachian region were closer to 1:1 line in all four products. Counties in Piedmont region had more evenly distributed biomass values in all products except the Blackard map. Counties in Eastern Shore region were more clustered, ranging between 40.1 and 79.2 Mg ha^−1^ for mean, and 4.6 and 7.8 Tg for total biomass respectively. Despite the improved correlation, the MBE was high in all four products, ranging between −33.0 and −54.6 Mg ha^−1^ for mean, and −3.5 and −5.8 Tg for total biomass respectively.

**Fig. 12:**
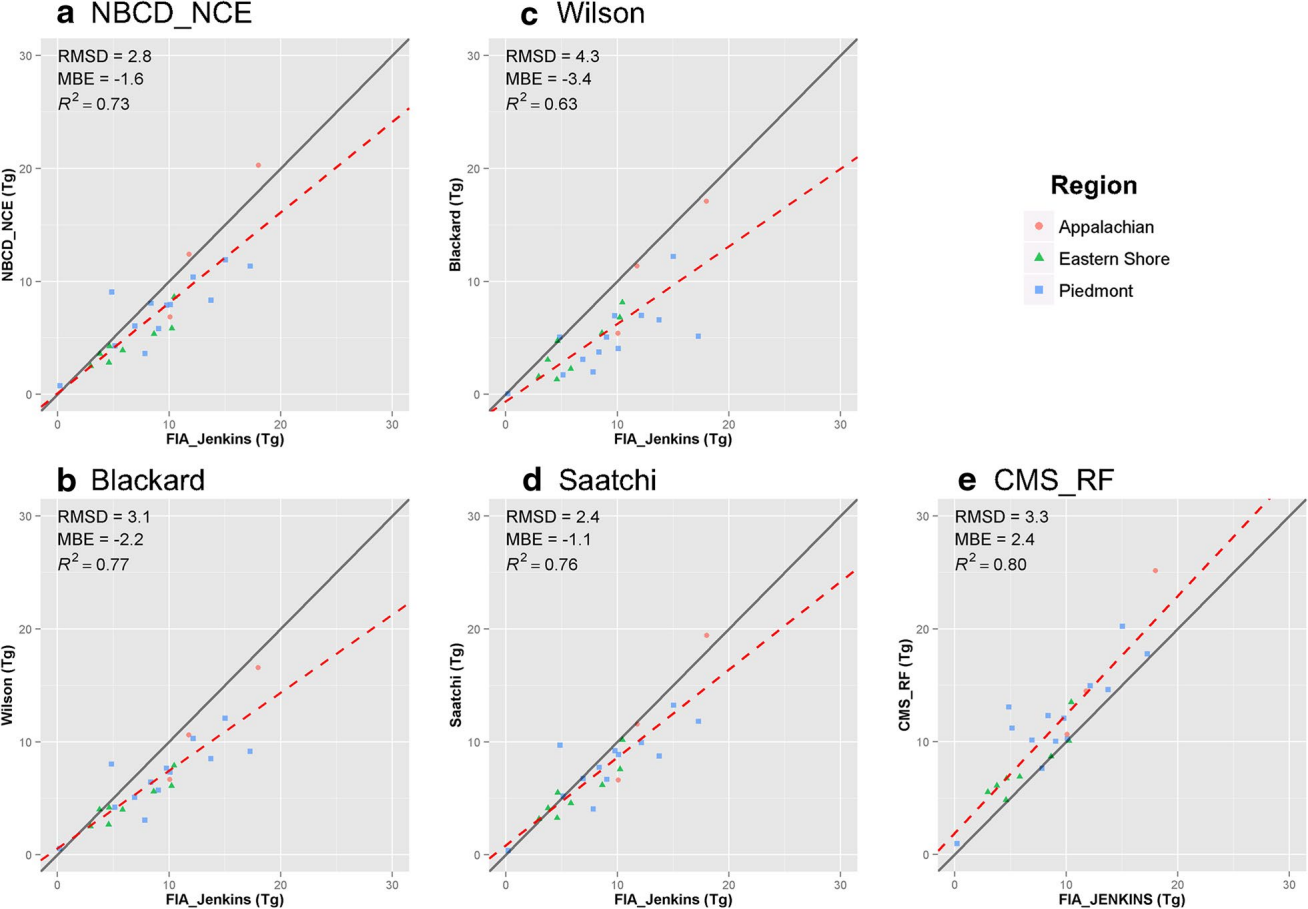
Scatter plots of county-level total biomass of four national products and CMS_RF against estimates from FIA_Jenkins. **a** NBCD_NCE at 30 m spatial resolution, **b** Blackard at 250 m spatial resolution, **c** Wilson at 250 m spatial resolution, **d** Saatchi at 250 m spatial resolution, and **e** CMS_RF at 30 m spatial resolution. The *x* axis represents the biomass totals from FIA_Jenkins, and the *y* axis represents corresponding national products in each plot. *Red dashed line* is the fitted regression line, and *gray solid line* is the 1:1 line. *FIA_Jenkins* represents biomass estimates using Jenkins allometrics and gap-filled for non-forest biomass.

County totals from the continental scale maps and the CMS_RF map were also compared with FIA totals (Fig. 12). For this comparison, we used the gap-filled Jenkins estimate from FIA data as it includes non-forested biomass [9]. Continental scale maps were strongly correlated with FIA at county level and had high coefficients of determination (0.63–0.80), but consistently underestimated biomass with a negative bias, ranging between −3.4 and −1.1 Tg for total biomass (Fig. 12a–d). The CMS_RF map showed good agreement too but had a positive bias and overestimated biomass, particularly in counties that had many low biomass areas such as in the Piedmont (Fig. 12e).

### Comparisons at the state level

**Fig. 13:**
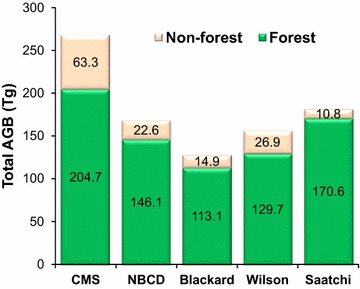
Comparison of total biomass at the state level from the CMS_RF map and the four national products over forested and non-forested areas. The forest/non-forest mask is aggregated from NLCD2006 with a threshold of 20 percentage.

There were significant differences between the biomass totals at the state level (Fig. 13). The national maps estimated state totals between 126.0 and 170.6 Tg and seemed to converge but were much lower when compared to the CMS_RF map. A detailed breakdown of mean and total biomass from all the maps is provided in Tables 3 and 4. The CMS_RF had higher mean (Tables 3, 4) and total biomass values (Fig. 13) over both forested and non-forested regions. The CMS_RF map also had higher total biomass than what is traditionally reported by FIA (164 Tg, 2008–2012 collection period) (Additional file 3: Table S1, [27]). However, we note that FIA does not measure trees in areas defined as “non-forest” and the allometric approach used by FIA to calculate tree biomass is known to give lower estimates in this region [9]. Adjusting for these nuances in the FIA data achieved better agreement with CMS_RF, although the FIA estimate was still lower by 43 Tg (Additional file 3: Table S1, [28]).

**Table 3:** Mean and total biomass for CMS_RF and NBCD_NCE products at 30 m resolution by forest and non-forest class

Type	CMS_RF		NBCD_NCE		Area compared^a^ (km^2^)
	Mean (Mg ha^−1^)	Total (Tg)	Mean (Mg ha^−1^)	Total (Tg)	
Forest	175.8	204.7	125.5	146.1	11,642
Non-forest	46.3	63.3	16.6	22.6	13,670
All	105.9	268.0	66.7	168.8	25,312
	Mg ha^−1^	Tg	Mg ha^−1^	Tg	km^2^

^a^Summarized from CMS_RF and NBCD_NCE products at 30 m resolution.

**Table 4:** Mean and total biomass for three national products at 250 m resolution by forest and non-forest class

Type	Blackard		Wilson		Saatchi		Area compared (km^2^)
	Mean (Mg ha^−1^)	Total (Tg)	Mean (Mg ha^−1^)	Total (Tg)	Mean (Mg ha^−1^)	Total (Tg)	
Forest	97.2	113.1	111.4	129.7	146.6	170.6	11,642
Non-forest	10.9	14.9	19.7	26.9	7.9	10.8	13,670
All	50.6	128.0	61.9	156.6	71.7	181.5	25,312
	Mg ha^−1^	Tg	Mg ha^−1^	Tg	Mg ha^−1^	Tg	km^2^

**Fig. 14:**
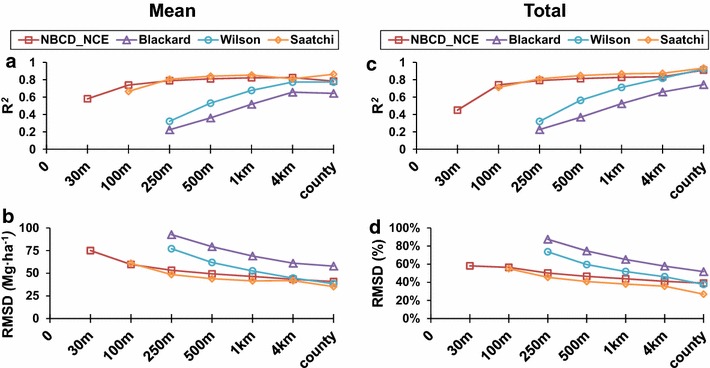
Trend in R^2^ and RMSD values among maps as a function of resolution. **a**, **b** Are Values for mean biomass density, while **c**, **d** are values for total biomass.

Lastly, we examined the coefficient of determination and corresponding errors as a function of resolution to detect trends and convergence between the maps (Fig. 14). The R^2^ values for both total and mean biomass increased with decreasing resolution, gradually moving closer to 0.90. Correspondingly, RMSD values decreased gradually stabilizing at ~35 Mg ha^−1^. The NBCD_NCE, Saatchi, and Wilson maps converged with near perfect agreement at around 4 km and onward. The Blackard map showed similar trends but less convergence with other products. Despite the improved agreement, the maps did not converge with the CMS_RF map at any scale considered in this study.

### Discussion

Spatial patterns of similarities and differences were consistent with land cover and physiographic gradients. Geographically, the greatest spatial discrepancies were in the Piedmont region. This is not unexpected, given the urban development and suburban sprawl in the region. Coarse scale maps did not capture the heterogeneity of urban-suburban landscapes as finely as the CMS_RF map, hence the difference (Fig. 3). Distinct spatial patterns of differences were also observed in Western Maryland and Southern Piedmont. These areas corresponded to dense forests where estimates from all the continental scale maps were lower. The Eastern shore had fewer discrepancies, probably because of sparse tree cover and lower biomass. However, unusually high values were noted in the national maps over several low-lying areas. This could be because of the mixed reflectance of water and vegetation over wetlands that is not easily separated in coarse resolution imagery [29].

We expected the 30 m NBCD_NCE map to be most similar to the CMS_RF map because it closely matched the spatial patterns in the CMS_RF map and had finer details than the other maps. However, the enhanced Saatchi map agreed more closely (Figs. 8, 4), despite having a coarse resolution (~90 m) and fewer predictions beyond 250 Mg ha^−1^. This was probably because the Saatchi map had more predictions in the 50–100 Mg ha^−1^ range than the NBCD_NCE map. The NBCD_NCE map had many pixels with very low biomass values (Fig. 9) which reduced its overall agreement with the CMS_RF map. Another surprising digression was the 250 m Wilson map that had a higher overall similarity index (FNI) than the NBCD_NCE map and the best agreement (Table 2) with the CMS_RF map over non-forested regions. A closer examination revealed that the Wilson map had better predictions in non-forested areas than any other map because it did not include a forest/non-forest mask and was developed using different models for areas greater than and less than 50 % NLCD forest cover. Thus, the agreement of continental scale maps with high-resolution estimates is not necessarily a function of spatial resolution but depends more on modeling approaches, time-lags and forest/non-forest definitions.

Choice of statistical/modelling approach was less critical in the CMS_RF estimation [18] but affected biomass predictions in other maps. The Blackard and Wilson maps used similar inputs yet had entirely different spatial distributions and histograms (Fig. 9) because of the difference in the regression models (Table 1). Similarly, we noted a strong influence of the MaxEnt model in the form of stratified predictions (Additional file 1: Figure S1) from the original Saatchi map. Such discrepancies are not easily detected in a broad comparison but are evident in a pixel-by-pixel comparison, as demonstrated in this study.

Continental scale maps (except the Wilson map) did not predict values outside forested areas because of limited FIA field plots for model development. This reduced their total biomass estimates and increased pixel-level discrepancies with the CMS_RF map. While we acknowledge that a fair comparison cannot be made over non-forested regions, we quantified the effect of excluding non-forest biomass on county and state level totals. Our results indicate that the underestimation is non-trivial, particularly in heterogeneous landscapes such as our study area. We provide further corroboration to findings of [10] and support the need for including biomass outside forests in carbon reporting.

Some apparent non-forested biomass values crept into the national map totals (Fig. 13) because of time lags between maps and inconsistencies in forest/non-forest masks from the NLCD product. We noticed high values (greater than 100 Mg ha^−1^) in non-forest histograms from some national maps (Fig. 9). These could be artifacts of forested areas that were converted since the production of the maps or differences in NLCD classifications over time. Some non-forested biomass was a result of edge effects in the coarse scale maps. Discrepancies could also be attributed to canopy cover thresholds used for comparisons (e.g. 20 % in this study). Larger thresholds can lead to lower non-forest biomass and vice versa. Some of these inconsistencies can be reduced by including sub-pixel estimates of tree cover [30] instead of a forest/non-forest mask in future continental scale mapping projects similar to the Wilson map [15]. This may greatly improve the agreement among maps, particularly in the 0–250 Mg ha^−1^ range (Fig. 8).

Another important difference between the continental scale maps and CMS_RF map was in high biomass forests. Continental scale maps had few predictions greater than 250 Mg ha^−1^. This was because they were developed using passive multispectral/radar data that were not sensitive enough to canopy structure in medium to high biomass ranges [31]. We expected some improvement in the enhanced Saatchi map as it included space-borne lidar data but did not observe any. This was probably because lidar data were used for model calibration rather than prediction. Biomass predictions were therefore influenced more by the 2D remote sensing data than the lidar inputs. One way of improving estimates beyond the 250 Mg ha^−1^ range is by including lidar measurements with higher resolution such as those from GEDI (expected launch in 2018) [32] or ICESAT-2 (expected launch in 2017) as predictor variables individually or through fusion with other datasets.

Some discrepancy in total biomass values between the different maps can be attributed to the differences in allometric models applied to the field dataset used to develop the maps. For example, re-calculating tree biomass from field data in Maryland with Jenkins equations [17] instead of the Component Ratio Method (CRM) [33] that is currently used by FIA, increases the total biomass by 11 %. Therefore, it is possible that the difference between the CMS_RF map and the maps derived from field data that applied the CRM (i.e., Blackard, Wilson, and Saatchi), could be somewhat lower than calculated in Table 4.

In general, the CMS_RF map had higher values than all the other maps because the discrete-return airborne lidar were effective in predicting biomass beyond 250 Mg ha^−1^ and the high-resolution tree cover mask ensured estimates for virtually all trees in the State. We noted some overestimation, particularly in the low biomass ranges, when we compared the CMS_RF map to FIA county totals (Fig. 12). This could be attributed to the Random Forests model that predicted higher biomass in areas with very low canopy height and cover or limitations with FIA estimates. More research is needed to understand these differences but the cross-validation of the CMS_RF map with FIA data at plot level was strong (Fig. 7) indicating the overall robustness of the CMS_RF map and its suitability as a reference for map comparisons.

Interestingly, all maps showed better agreement at the county scale despite large discrepancies at finer scales. One reason for this could be that all maps captured some of the variability in biomass as a function of canopy structure, land cover type and physiographic gradient, irrespective of inputs and modeling approaches. This was evident from similarities in spatial patterns and FNI values. All maps (except the Blackard map) had greater than 45 % similarity with the CMS_RF map at the pixel level, contributing to the agreement. Secondly, all continental scale maps were developed using statistical regressions with FIA data which is meant to provide an unbiased state-wide estimate. A regression model, by nature, estimates the mean of the predictor data. This is also applicable to the Random Forest model used to generate the CMS_RF map. Since all maps were more or less accurate in predicting mean biomass, there was a fundamental agreement despite the variability at fine scales. Outliers reduced on spatial aggregation and the agreement between maps increased, as observed from the decreasing RMSD and increasing goodness of fit (Fig. 14).

Continental scale maps showed increasing agreement at coarse scales and converged between 4 km and 10 km (county-scale). This is similar to trends observed in Mitchard et al. [4] and Avitabile et al. [26]. However, the agreement is misleading, as these maps do not converge with the CMS_RF map at any scale considered in this study (Fig. 14). The mismatch was because of a relatively constant negative bias in all the continental scale estimates when compared to the CMS_RF and was as high as 30 % (Figs. 10, 11) at county scale. The negative bias was primarily because of the underestimation in high biomass ranges and lack of predictions in non-forested areas. Since this difference does not diminish with coarsening resolution, we argue that local-scale discrepancies may affect carbon reporting at all levels and should not be ignored.

### Conclusion

A detailed validation with high-resolution estimates can be valuable in identifying discrepancies and making continental-scale maps truly applicable to carbon accounting applications. We demonstrated one example over temperate forested and non-forested landscapes in Maryland. More studies across different biomes are required to confirm these findings. Armed with a comprehensive understanding from such validations, we can improve and integrate multi-source datasets to inform carbon monitoring efforts.

## Additional files

Additional file 1:
**Figure S1.** Scatter plots of biomass at 250 m resolution from old Saatchi (v1) and new Saatchi (v2) maps versus CMS_RF product.
Additional file 2:
**Figure S2.** Histograms showing the distribution of forest biomass from old Saatchi (v1), new Saatchi (v2), and CMS_RF maps.
Additional file 3:
**Table S1.** Total Maryland biomass, in Tg, for CMS_RF at 30 m resolution and FIA calculations (2008–2012 cycle) by forest and non-forest classification. FIA forest and non-forest definitions do not follow NLCD landcover classes, rather by on the ground plot conditions. Non-forest biomass in the FIA dataset was calculated by methods described in [28].
