# Supplementary figures and images for: Local discrepancies in continental scale biomass maps: a case study over forested and non-forested landscapes in Maryland, USA
Source: Carbon Balance Manag. 2015 Aug 16;10:19. doi: 10.1186/s13021-015-0030-9 (PMC4537504; doi:10.1186/s13021-015-0030-9)

## Slide 1
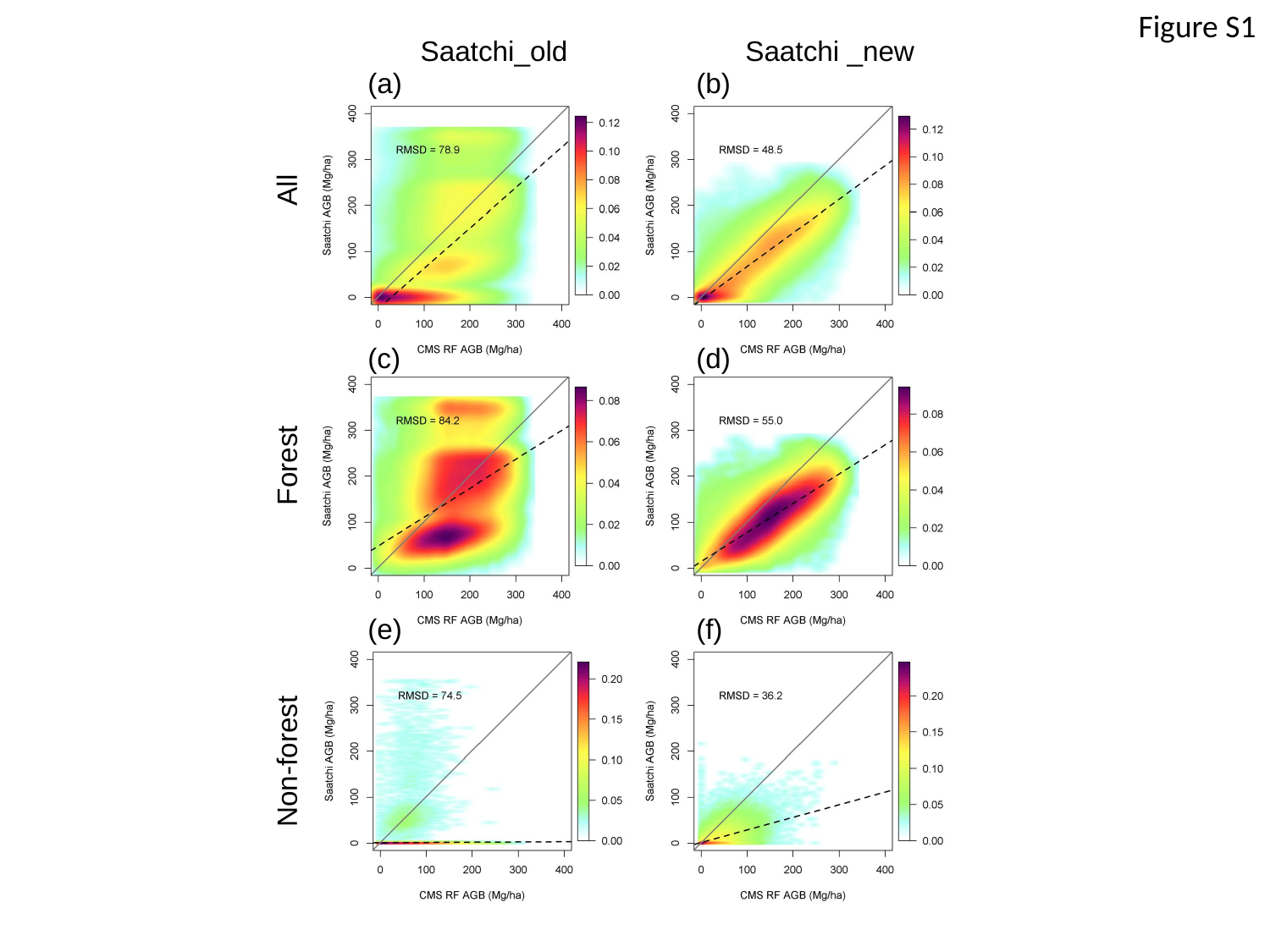

Figure S1
Saatchi_old
Saatchi _new
(a)
(b)
(c)
(d)
(e)
(f)
All
Forest
Non-forest

Supplement: Additional file 1: — Figure S1. Scatter plots of biomass at 250 m resolution from old Saatchi (v1) and new Saatchi (v2) maps versus CMS_RF product. [file 13021_2015_30_MOESM1_ESM.pptx]

## Slide 1
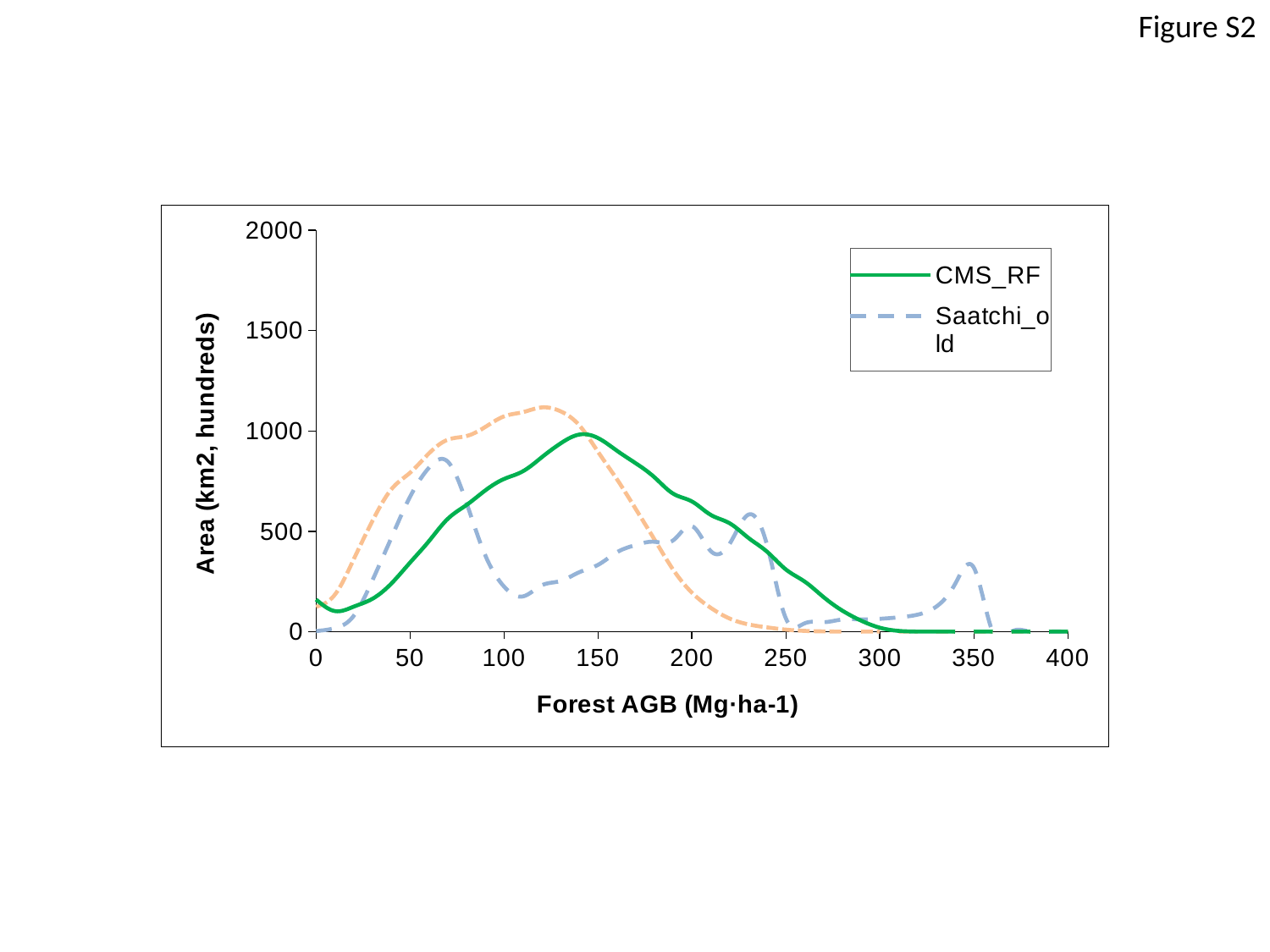

Figure S2
### Chart
| Category | CMS_RF | Saatchi_old | Saatchi_new |
|---|---|---|---|

Supplement: Additional file 2: — Figure S2. Histograms showing the distribution of forest biomass from old Saatchi (v1), new Saatchi (v2), and CMS_RF maps. [file 13021_2015_30_MOESM2_ESM.pptx]
