# Supplementary material for: Local discrepancies in continental scale biomass maps: a case study over forested and non-forested landscapes in Maryland, USA
Source: Carbon Balance Manag. 2015 Aug 16;10:19. doi: 10.1186/s13021-015-0030-9 (PMC4537504; doi:10.1186/s13021-015-0030-9)
Supplement: Additional file 3: — Table S1. Total Maryland biomass, in Tg, for CMS_RF at 30 m resolution and FIA calculations (2008–2012 cycle) by forest and non-forest classification. FIA forest and non-forest definitions do not follow NLCD landcover classes, rather by on the ground plot conditions. Non-forest biomass in the FIA dataset was calculated by methods described in [28]. [file 13021_2015_30_MOESM3_ESM.docx]

Table S1. Total Maryland biomass, in Tg, for CMS_RF at 30 m resolution and FIA calculations (2008-2012 cycle) by forest and non-forest classification. FIA forest and non-forest definitions do not follow NLCD landcover classes, rather by on the ground plot conditions. Non-forest biomass in the FIA dataset was calculated by methods described in [[28](#_ENREF_28)].

|  | **CMS_RF** | **CRM* + NF** | **Jenk. + NF** |
| --- | --- | --- | --- |
| Forest | 204.7 | 163.9** | 182.8 |
| Non-forest | 63.3 | 25.5 | 28.5 |
| **All** | **268.0** | **189.4** | **211.3** |

*CRM refers to the Component Ratio Method for calculating biomass [[31](#_ENREF_31)].

**The original and official biomass estimate reported by FIA using the EVALIDATOR tool [[27](#_ENREF_27)].
